# Chronic hepatitis caused by persistent parvovirus B19 infection

**DOI:** 10.1186/1471-2334-10-246

**Published:** 2010-08-20

**Authors:** Trine H Mogensen, Jens Magnus B Jensen, Stephen Hamilton-Dutoit, Carsten S Larsen

**Affiliations:** 1 Department of Infectious Diseases, Aarhus University Hospital, Skejby, DK-8200 Aarhus N, Denmark; 2 Department of Clinical Immunology, Aarhus University Hospital, Skejby, DK-8200 Aarhus N, Denmark; 3 Department of Pathology, Aarhus University Hospital, Aarhus Sygehus, DK-8000 Aarhus C, Denmark

## Abstract

**Background:**

Human infection with parvovirus B19 may lead to a diverse spectrum of clinical manifestations, including benign erythema infectiosum in children, transient aplastic crisis in patients with haemolytic anaemia, and congenital hydrops foetalis. These different diseases represent direct consequences of the ability of parvovirus B19 to target the erythroid cell lineage. However, accumulating evidence suggests that this virus can also infect other cell types resulting in diverse clinical manifestations, of which the pathogenesis remains to be fully elucidated. This has prompted important questions regarding the tropism of the virus and its possible involvement in a broad range of infectious and autoimmune medical conditions.

**Case Presentation:**

Here, we present an unusual case of persistent parvovirus B19 infection as a cause of chronic hepatitis. This patient had persistent parvovirus B19 viraemia over a period of more than four years and displayed signs of chronic hepatitis evidenced by fluctuating elevated levels of ALAT and a liver biopsy demonstrating chronic hepatitis. Other known causes of hepatitis and liver damage were excluded. In addition, the patient was evaluated for immunodeficiency, since she had lymphopenia both prior to and following clearance of parvovirus B19 infection.

**Conclusions:**

In this case report, we describe the current knowledge on the natural history and pathogenesis of parvovirus B19 infection, and discuss the existing evidence of parvovirus B19 as a cause of acute and chronic hepatitis. We suggest that parvovirus B19 was the direct cause of this patient's chronic hepatitis, and that she had an idiopathic lymphopenia, which may have predisposed her to persistent infection, rather than bone marrow depression secondary to infection. In addition, we propose that her liver involvement may have represented a viral reservoir. Finally, we suggest that clinicians should be aware of parvovirus B19 as an unusual aetiology of chronic hepatitis, when other causes have been ruled out.

## Background

Human infection with parvovirus B19 may lead to a diverse spectrum of clinical manifestations, including benign erythema infectiosum in children, transient aplastic crisis in patients with haemolytic anaemia, and congenital hydrops foetalis. These different diseases represent direct consequences of the ability of parvovirus B19 to target the erythroid cell lineage. Recently however, parvovirus B19 infection has been associated with diseases involving other cell types. This has prompted important questions regarding the tropism of the virus and its possible involvement in the pathogenesis of a broad range of medical conditions, including idiopathic arthritis [1], vasculitis [2], meningoencephalitis [3], hepatitis [4], and myocarditis [5]. Here, we present an unusual case of chronic hepatitis in a patient with persistent parvovirus B19 infection. We describe the current knowledge on the pathogenesis and clinical manifestations of parvovirus B19 infection and discuss the evidence of parvovirus B19 as a cause of acute and chronic hepatitis.

## Case presentation

A previously healthy 50-year old female was referred to the outpatient clinic of a general hospital due to excessive fatigue and exanthema. Five month prior to the evaluation, she had an episode of febrile illness with influenza-like symptoms followed by the appearance of an exanthema. At that time, a dermatologist made a presumptive diagnosis of vasculitis and prescribed prednisolone, after which the exanthema had subsided. There had been no neurological, cardiopulmonary or gastrointestinal symptoms, and no arthralgias, weight loss or fever. The patient did not have any history of alcohol or intravenous drug abuse and did not drink any alcohol throughout her disease. The physical examination was unremarkable.

Routine blood tests revealed slight lymphopenia (0.86 × 10^9^/L) and thrombocytopenia (122 × 10^9^/L) but no anaemia (haemoglobin 8.5 mmol/L). In addition, modest elevations of liver enzymes were noted with ALAT of 57 U/L as well as normal tests of renal function. Markers of inflammation, such as CRP and ESR, were within the normal range.

An infectious aetiology of the patients' symptoms was suspected. Hepatitis A, B, and C, HIV, and borrelia serologies were all negative. In addition, PCR for HIV- and HCV-RNA were negative. Antibody titres for EBV, CMV, HHV6, parvovirus B19 and toxoplasmosis were IgG positive but IgM negative. However, PCR for parvovirus B19 DNA in serum was positive, suggesting chronic parvovirus B19 infection. In order to exclude the possibility of autoimmune disease, ANA, ANCA, rheumatoid factor, and anti-phospholipid antibodies were evaluated and were all negative. Moreover, smooth-muscle cell antibody and mitochondrial antibody were negative, excluding autoimmune hepatitis and primary biliary cirrosis, respectively. Finally, coeruloplasmin, s-ACE, and s-ferroxidase were also analysed to rule out other aetiologies of hepatic damage.

**Figure 1:**
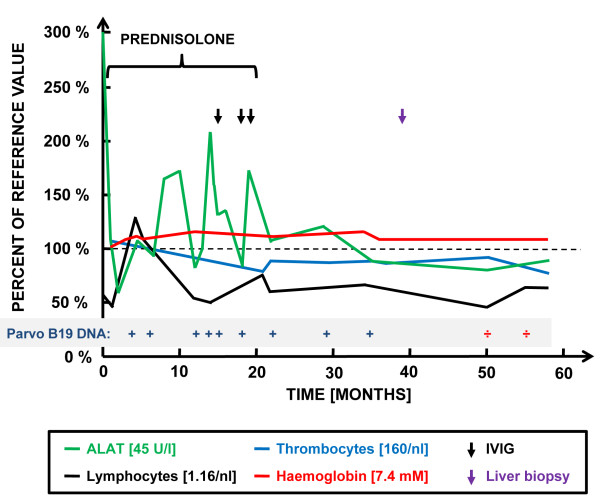
**Time course of hepatic enzymes and haematological, immunological, and virological parameters**. Values are shown as percentages of reference values over time (t = 0: time of initial symptoms). Reference values were chosen as the lowest of the normal range, with the exception of ALAT, for which the highest normal range value was used. Time points for intravenous immunoglobulin (IVIG), prednisolone treatment, liver biopsy, and parvovirus B19 DNA status are indicated.

Due to persistent symptoms and positive parvovirus B19 DNA in serum, the patient was evaluated for an immunodeficiency. The CD4 positive T cell count was reduced (261-330 cells/mL), but further analysis demonstrated equally reduced T lymphocyte numbers in all subpopulations, including CD4- and CD8- positive subsets. Additionally, a markedly reduced B lymphocyte and NK cell count was found, although with normal NK cell function. The serum concentrations of IgA, IgM and IgG, including IgG subclasses, were all normal. These immunological and haematological values are depicted in figure 1.

The patients' symptoms of fatigue, fluctuating elevations of ALAT, and moderate lymphopenia were unaltered for more than one year with persistent evidence of parvovirus DNA in serum measured by PCR every 6 months. A therapeutic trial was made with high-dose intravenous immunoglobulin infusions for five days and repeated twice over as period of 5 months. Unfortunately, the treatment did not result in the clearance of parvovirus B19 DNA from serum.

**Figure 2:**
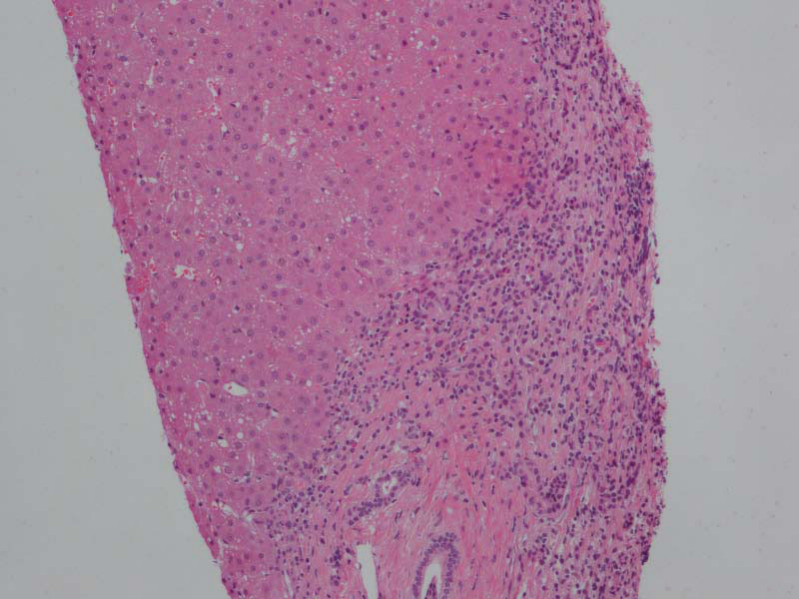
**Liver biopsy with chronic hepatitis**. An expanded portal tract shows chronic inflammation with a modest degree of active interface hepatitis. On the upper right hand side of the figure is an area of bridging septal fibrosis. (Haematoxylin and eosin staining).

Due to persistently elevated levels of ALAT, a liver biopsy was performed three years after the onset of the illness. Histology demonstrated slight to moderate portal chronic inflammation with focal interface hepatitis as well as portal and periportal fibrosis with scattered foci of bridging fibrosis. A histopathological diagnosis of chronic hepatitis was made (Figure 2).

Four years after the debut of symptoms, parvovirus B19 DNA in serum for the first time was undetectable. Notably, the level of ALAT also was normal. However, her lymphopenia and thrombocytopenia persisted, although it was less pronounced (Figure 1). At that time, the patient reported being well and slowly regaining her normal level of activity as prior to her parvovirus B19 infection. Immunological evaluation after one more year demonstrated persistent lymphopenia (330 cells/mL) and thrombocytopenia (220 cells/mL) but normal NK cell function.

## Discussion

Human parvovirus B19 infection is frequent with most infections occurring during childhood and as much as 65% of the adult population affected as evidenced by seropositivity [4]. The natural history of parvovirus B19 infection in humans is biphasic with an initial febrile state accompanied by non-specific influenza-like symptoms due to viral replication in the upper respiratory tract and spread by viraemia to the bone marrow. The virus enters erythroid progenitor cells through the erythrocyte blood group P antigen and subsequently replicates and establishes lytic infection. The second symptomatic stage of the illness, which is immune mediated, is precipitated by the appearance of virus-specific IgM antibodies, inhibition of viraemia, and formation of immune complexes responsible for a maculopapular exanthema and polyarthritis. In children, the classical picture of erythema infectiosum is a distinctive rash on the cheeks, whereas in adults, polyarthritis dominates and may be preceded by a universal rash. Viral DNA is typically present in serum up to 6 months after onset of symptoms [6]. Therefore, detection of viral DNA later than this time is suggestive of persistent infection.

Several studies have implicated parvovirus B19 in various different types of pathology of the liver. First, several reports have suggested a pathogenic role for parvovirus B19 in the development of acute hepatitis [7-9] and fulminant liver failure of unknown aetiology [10,11]. Furthermore, persistence of B19 DNA in liver and bone marrow has been associated with acute liver disease and aplastic anaemia [9,12,13]. Furthermore, interest has focused on a possible effect of co-infection with parvovirus B19 on the natural history of chronic hepatitis B and C. By examining serum samples from patients, Hsu et al. found that parvovirus B19 DNA was frequently present in patients with chronic hepatitis B and C, indicating that parvovirus B19 may not be eradicated in these patients [14]. However, co-infection of parvovirus B19 with hepatitis C virus (HCV) or hepatitis B virus (HBV) did not increase the frequency of liver dysfunction [14]. This is in contrast to another study, in which a significant correlation between parvovirus B19 co-infection and a greater likelihood of progression to more severe HBV-associated liver disease was reported in Vietnamese patients [15]. Finally in a study involving European patients, intrahepatic long-term persistence of parvovirus B19 in both end-stage liver tissue and routine biopsies was demonstrated, but parvovirus B19 DNA was only very rarely detected in serum samples from hepatitis B and C patients, and no evidence was found for parvovirus B19 as a factor worsening liver disease in chronic hepatitis C [16].

The precise role of parvovirus B19 as a bona fide hepatitis virus directly causing chronic hepatitis remains controversial, but based on the paucity of cases reported in the literature, this clinical manifestation seems to be very rare. One case described an immunocompetent male patient presenting with persistent fever, jaundice, polyarthritis, and evidence of persistent B19 infection [4]. Another case was described by Pinho et al., who reported the presence of active B19 infection in one patient with hepatitis among 129 cases analysed with non A-E hepatitis [17]. The patient, a 56-year old female patient with severe hepatitis and submassive necrosis by liver biopsy, was PCR positive for B19 DNA in blood as well as in liver tissue. However, the patient described by Pinho differs significantly from our patient, since an autoimmune cause could not be ruled out, prompting treatment with prednisolone and azathioprin [17]. The patient we describe here was also persistently B19 DNA positive in blood by PCR and her liver biopsy taken after approximately 3 years of active infection displayed signs of chronic inflammation and moderate fibrosis. Furthermore, alternative infectious and non-infectious causes of chronic hepatitis and liver pathology were ruled out. We therefore believe that her chronic hepatitis was caused by persistent parvovirus B19 infection. This is supported by normalization of ALAT after clearance of parvovirus B19 from blood. Importantly, viral clearance could not be attributed to immunoglobulin treatment, since it happened at least three years later as illustrated in figure 1. Immunoglobulin infusions have been reported to be successful in treating chronic parvovirus B19 infection, particularly in immunocompromised individuals, but in the absence of randomized trials, no evidence or consensus has been reached [18].

The mechanism by which parvovirus B19 induces liver pathology and failure remains unknown. *In vitro *parvovirus B19 is able to enter hepatocytes through binding to the P antigen (globoside) [19], although hepatocytes are assumed to be non-permissive for parvovirus replication [20]. One mechanism suggested for B19-induced hepatopathy include effects of the viral protein non-structural protein (NS) 1 through activation of interleukin-6 expression [21]. More recently, Poole et al. demonstrated parvovirus B19-induced apoptosis of hepatocytes induced by NS1 and mediated through an intrinsic caspase pathway, involving caspases 3 and 9 [22,23]. Indeed, authors in this field have suggested that it should be further investigated, whether intense liver involvement predisposes or correlates with persistent parvovirus B19 infection [4]. However, given that the prevalence of parvovirus B19 in healthy tissue remains unknown, it is difficult to demonstrate the role of parvovirus B19 in the aetiology of hepatitis, since the mere presence of viral DNA in tissue can not be used to infer causality [13,16]. Thus, whether parvovirus B19 is a pathogenic agent of fulminant liver failure and non A-E hepatitis, a risk factor accelerating liver dysfunction due to other agents, or alternatively a bystander with no influence on liver pathology, is still unresolved.

Parvovirus B19 infection was previously considered a cause of chronic infection only in immunocompromised individuals, in whom symptomatic B19 infection and anaemia may persist for months or even years [24,25]. More recently, the existence of a chronic type of infection associated with continuous virus production has been established, and this clinical entity may occur also in apparently immunocompetent individuals [4]. This raises the question, as to whether our patient may have been immunocompromised prior to her parvovirus B19 infection, or whether the observed lymphopenia was merely a result of longstanding chronic viral infection, which may per se cause immunosuppression [26]. In addition, prednisolone treatment may have caused a degree of immunosuppression. The patient exhibited decreases in T lymphocyte and NK cell populations, which were present early in the course of disease and persisted for years after clearance of parvovirus B19 infection, suggesting that they were not secondary to infection but rather predisposed her to chronic parvovirus B19 infection. It is notable, however, that she did not at any time develop anaemia, which might have been suspected. [27,28].

## Conclusions

In most cases, Parvovirus B19 infection causes relatively benign self-limiting disease. However, accumulating evidence suggests that this virus can also affect other cell types resulting in diverse clinical manifestations, of which the pathogenesis remains to be fully elucidated. Here, we have described an unusual case of persistent parvovirus B19 infection as a cause of chronic hepatitis [27,28]. The patient described here developed persistent infection despite the presence of normal humoral immune competence and did not have anaemia but rather had hepatic involvement as the primary manifestation. This may suggest that the liver represented the main viral reservoir of this patient's persistently active infection. Finally, it cannot be ruled out that her idiopathic lymphopenia may have predisposed her to persistent infection. Clinicians should be aware that parvovirus B19 may be a rare cause of hepatic pathology, when other causes of hepatitis have been ruled out.

## Competing interests

The authors declare that they have no competing interests.

## Authors' contributions

THM and CSL identified the patient and did the "literature search". JMBJ interpreted the immunological data and prepared figure 1. SHD performed the histopathological analysis of the liver biopsy and prepared figure 2. THM prepared the manuscript and all authors read and approved the final version of the manuscript.

## Pre-publication history

The pre-publication history for this paper can be accessed here:

http://www.biomedcentral.com/1471-2334/10/246/prepub
